# Supplementary material for: Addressing the ADME Challenges of Compound Loss in a PDMS-Based Gut-on-Chip Microphysiological System
Source: Pharmaceutics. 2024 Feb 20;16(3):296. doi: 10.3390/pharmaceutics16030296 (PMC10974294; doi:10.3390/pharmaceutics16030296)
Supplement: Supplementary file 1 [file pharmaceutics-16-00296-s001.zip › pharmaceutics-2855861-supplementary.pdf]

Article

Addressing the ADME Challenges of Compound Loss in a PDMS-based Gut-On-Chip Microphysiological System

Patrick Carius, Ferdinand Anton Weinelt, Chris Cantow, Markus Holstein, Aaron M. Teitelbaum and Yunhai Cui *

Boehringer Ingelheim Pharma GmbH & Co KG, Dept. Drug Discovery Sciences, Biberach, Germany, patrick.carius@boehringer-ingelheim.com (P.C.); ferdinand_anton.weinelt@boehringer-ingelheim.com (F.A.W.); chris.cantow@boehringer-ingelheim.com (C.C.); markus.holstein@boehringer-ingelheim.com (M.H.); aaron.teitelbaum@boehringer-ingelheim.com (AT); yunhai.cui@boehringer-ingelheim.com (Y.C.)

* Correspondence: yunhai.cui@boehringer-ingelheim.com; Tel.: +49-7351-54-92193

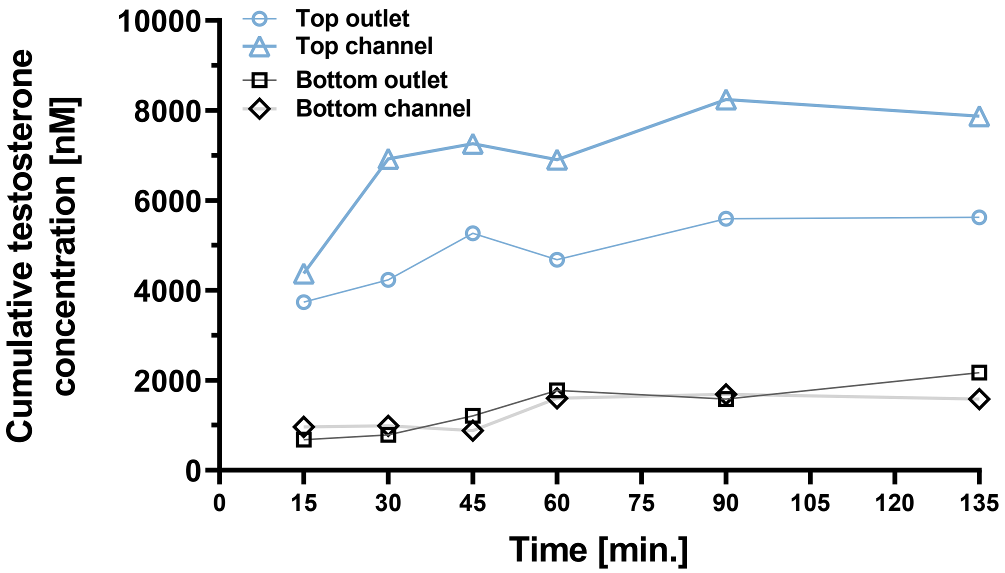

Supplementary Figure S1. Concentration over time profiles of testosterone perfused into the top inlet at 200 µL/h at a concentration of 10 µM in an Emulate organ chip from the compound distribution kit.

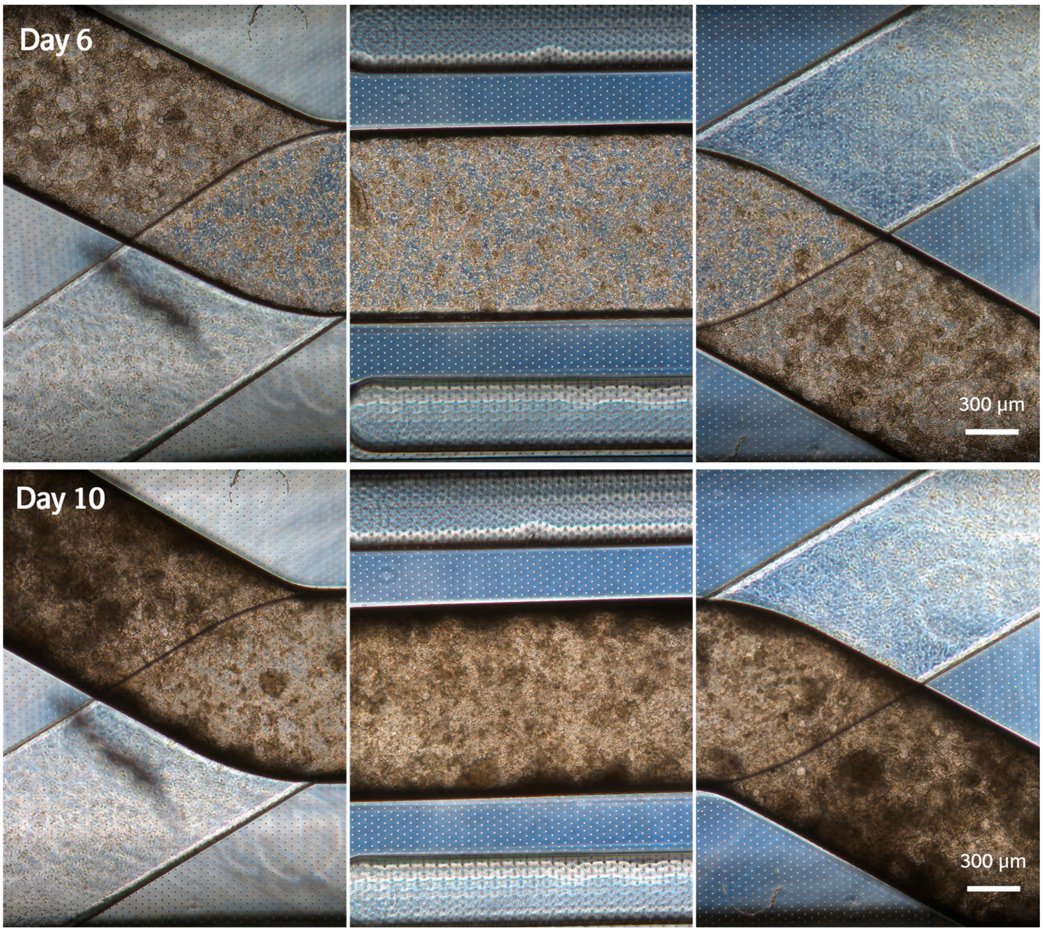

Supplementary Figure S2. Microscopic photographs of Caco-2/HUVEC gut-on-chip on day 6 and day 10.

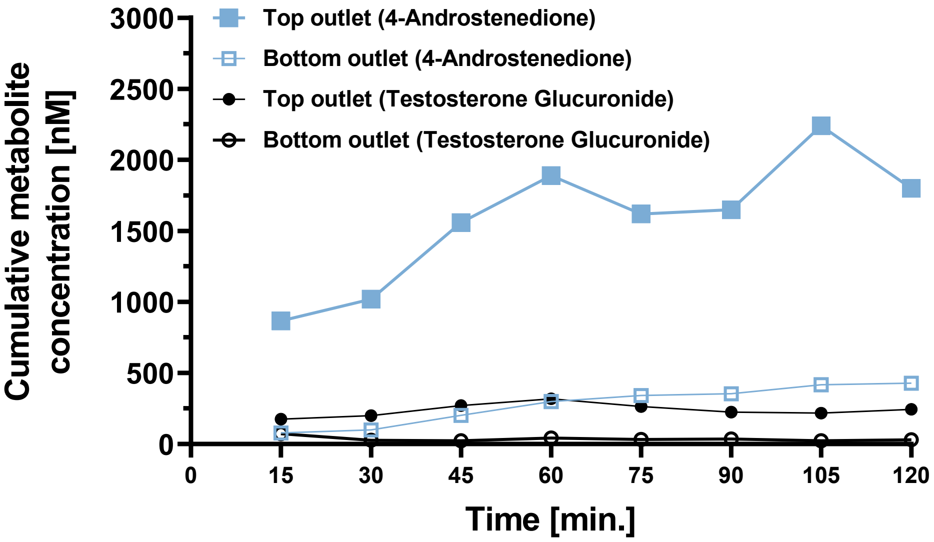

Supplementary Figure S3. Concentration over time profiles of testosterone metabolites in Caco-2/HUVEC gut-on-chips perfused with Testosterone into the top inlet at 200 µL/h at a concentration of 10 µM.

Supplementary Table S1. Compound loss of Testosterone metabolites in cell-free organ-chips.

	absorption after 1h [%]		
	Bottom channel	Top channel	**Combined**
**Metabolite**	mean	mean	mean
4-Androstenedione	70	65	**68**
6β-Hydroxytestosterone	0	0	**0**
Testosterone glucuronide	26	29	**28**

Supplementary Table S2. Inhibition of P-gp mediated Quinidine efflux in MDCK-MDR1 cells by Elacridar

Elacridar concentration (μM)	Quinidine efflux
0	32
0.2	3.9
1	1.7
5	0.7
